# The modulation of facial mimicry by attachment tendencies and their underlying affiliation motives in 3-year-olds: An EMG study

**DOI:** 10.1371/journal.pone.0218676

**Published:** 2019-07-01

**Authors:** Stefania V. Vacaru, Johanna E. van Schaik, Sabine Hunnius

**Affiliations:** 1 Donders Institute for Brain, Cognition, and Behaviour, Radboud University, Nijmegen, The Netherlands; 2 Vrije Universiteit, Amsterdam, The Netherlands; Leiden University, NETHERLANDS

## Abstract

From early in life, facial mimicry represents an important example of implicit non-verbal communication. Facial mimicry is conceived of as the automatic tendency to mimic another person’s facial expressions and is thought to serve as a social glue among interaction partners. Although in adults mimicry has been shown to be moderated by the social context and one’s needs to affiliate with others, evidence from behavioural mimicry studies suggest that 3-year-olds do not yet show sensitivity to social dynamics. Here, we examined whether attachment tendencies, as a proxy for interindividual differences in affiliation motivation, modulates facial mimicry in 3-year-olds. Resistant and avoidant insecure attachment tendencies are characterized by high and low affiliation motivation, respectively, and these were hypothesized to lead to either enhancement or suppression of mimicry. Additionally, we hypothesized that these effects will be moderated by inhibitory control skills. Facial mimicry of happy and sad expressions was recorded with electromyography (EMG), attachment tendencies were assessed with a parent-report questionnaire and inhibitory control with the gift delay task. The final sample consisted of 42 children, with overall scores suggesting secure attachment. Our findings revealed that 3-year-olds mimicked happy and sad facial expressions. Moreover, resistant tendencies predicted enhanced sad but not happy facial mimicry, whereas avoidant tendencies were not significantly related to mimicry. These effects were not moderated by inhibitory control skills. In conclusion, these findings provide the first evidence for the modulation of mimicry by attachment tendencies and their underlying motivation for affiliation in young children, specifically for negatively-valenced emotional expressions.

## Introduction

Throughout development, children experience a myriad of social interactions which form the basis for their affective ties. Already from the first days of life, children engage in one of the simplest, yet most essential type of social interaction, namely face-to-face interactions [1, 2]. These are organized around playful exchanges of vocalizations and facial expressions [3], in which parents and infants imitate each other [4, 5]. The imitation of facial expressions—commonly referred to as facial mimicry—is thought to be an automatic process during social encounters [6–12]. Moreover, it has been argued that early face-to-face interactions may form the basis of facial mimicry [13]. Although newborns can perceptually discriminate facial emotional expressions [14], the earliest evidence of emotional facial mimicry in infancy has been found around 5 months of age [15], but not earlier [16]. Importantly, scholars have described the function of mimicry as a “social glue” fostering liking, similarity and affiliation among social partners [17–19] Indeed, facial mimicry of emotional expressions is thought to facilitate emotion recognition [20] and be related to empathy [21, 22].

In recent years, several studies have addressed the emergence and development of various forms of mimicry (e.g. facial, behavioural) throughout infancy [23–25] and childhood [8, 26]. Altogether, these findings indicate that the general propensity to mimic is present in young children. However, while the adult literature has focused on mimicry’s important social functions [27–31], only a handful of studies have examined the social sensitivity of mimicry during childhood. Developmental evidence by van Schaik and Hunnius [32] showed that young children preferentially mimic in-group versus out-group members and found that only 4- to 6-year-olds mimicked their in-group members more than the out-group members, whereas 3-year-olds did not. Correspondingly, the older children expressed an in-group preference, suggesting that the motivation to affiliate with in-group members might have contributed to their socially-selective mimicry. However, these findings remain inconclusive as to whether the 3-year-olds displayed indiscriminate mimicry because of a lack of regulatory cognitive skills or due to the complexity of the manipulation used to elicit group membership [26, 32]. Relatedly, higher cognitive skills, such as inhibitory control, have been argued to be a potential mechanism enabling selective mimicry (i.e. whom and when to mimic). Indeed, in another study, the authors demonstrated that 5-year-olds’ selective mimicry towards an unkind versus kind experimenter is modulated by inhibitory control skills [33]. Together, these studies indicate that mimicry becomes increasingly sensitive to social dynamics throughout development, as higher cognitive skills are acquired.

Young children may lack sensitivity to complex social dynamics, yet they do not lack sensitivity in their intrinsic motivation to affiliate with others. Even though affiliating with others is primarily an inherent need, its strength can vary across the lifespan and across individuals [34, 35]. It has been argued that experiences in the early infant-parent relationships profoundly shape children’s motivation to form relationships with others [36] and future social-emotional development [37]. Indeed, infants form attachment bonds with their primary caregivers [38, 39] from which long-lasting attachment tendencies emerge [40, 41] that prevail in future relationships (e.g. relationships with peers, romantic or parenting relationships) [42–44]. Different attachment tendencies are characterized by different motivations for affiliation and strategies to attain it [45–48]. Particularly, organized and disorganized insecure attachment is classified based on behaviours during stressful circumstances (e.g. parental separation). Disorganized attachment entails a breakdown in the behavioural response to distress, characterized by fear and incoherent behavioural strategies [49–51]. On the contrary, organized insecure attachment presupposes a coherent behavioural coping strategy and has been shown to be characterized by two distinct patterns, namely avoidant and resistant-ambivalent. Children with an insecure avoidant attachment tendency are thought to deactivate the attachment system [52], to avoid intimate emotional relationships and to minimize proximity with others [53]. Instead, children with an insecure resistant attachment tendency are thought to hyperactivate the attachment system and maximize proximity and intimacy with others as means to seek confirmation about themselves [54, 55]. Particularly, the resistant attachment pattern is a complex strategy, as children almost compulsively seek proximity with others, although they then tend to not accept comfort during distress [56]. Despite their ambivalence between seeking contact, clinging and over-dependency on the one hand and refusing comfort during distress on the other, resistant-ambivalent children show a clear strategy to increase proximity to others. Consistent with this portrayal of insecure attachment, evidence shows that the resistant and the avoidant type are characterized by low and high affiliation motivation, respectively [57]. As reviewed above, one strategy to attain affiliation with others is mimicry. Indeed, some evidence shows that different attachment tendencies, as characterized by differences in the motivation to attain affiliations with others, modulate implicit mimicry in adults [58, 59]. Hall and colleagues found that adults’ insecure attachment yielded diminished behavioural mimicry (i.e. face-rubbing) [58]. Furthermore, evidence from an emotional facial mimicry study revealed that while non-avoidant individuals mimicked happy and angry facial expressions, the avoidant group tended to respond with a smile to the angry expressions [59]. The authors interpreted this response as an attempt to suppress negative emotions, due to emotion regulation difficulties of insecurely attached individuals. Altogether this evidence not only suggests that there is a relation between specific attachment patterns and mimicry, but also that attachment insecurity modulates facial mimicry of negative emotions. In sum, previous literature showed that facial mimicry emerges in infancy [15], but to date only the effect of experimentally-manipulated affiliation motivations on mimicry have been investigated in young children [32, 33]. Moreover, the understanding of complex social dynamics in 3-year-olds might be poorer than in older children and this may limit their ability to mimic selectively. Yet, the intrinsic motivation for affiliation stemming from early attachment relationships might already play an important role in modulating mimicry responses in younger children. Thus, in this study we examined whether attachment tendencies underlying different affiliation motives modulate facial mimicry and whether this relationship is moderated by inhibitory control. To this end, we measured facial mimicry in a group of 3-year-old children in response to happy and sad emotional facial expressions by means of subtle facial electromyographic (EMG) activation [60]. We used two examples of positive and negative emotions, similar to the previous study by Sonnby-Borgstrom and Jonsson [59], yet we chose sad instead of angry, given that angry expressions may elicit a fear reaction, rather than a mimicry response. Indeed, Geangu and colleagues [8] found a fear muscle activation (i.e. frontalis) in response to anger in 3-year-old children. Facial mimicry is described as the pattern of activation of a pair of muscles congruent with the observed facial expression morphology: facial mimicry of happy expressions, for instance, is the result of increased activation in the zygomaticus major muscle [7] and decreased activation in the corrugator supercilii muscle [61], whereas the reversed pattern of muscle activation corresponds to facial mimicry of sad facial expressions [62]. We assessed attachment tendencies as a proxy for affiliation motives, and we chose the resistant and the avoidant types for our investigation, as these entail two distinct organized behavioural strategies to attain affiliation. We disregarded the disorganized type in the current study, as it is not characterized by an organized set of behavioural strategies and thus no specific hypotheses can be formulated.

The aims of the current study were threefold. First, we investigated whether 3-year-olds mimic happy and sad facial expressions. To address this aim, we examined whether children would display increased ZM and decreased CS muscle activation in response to happy facial expression, and conversely, they would display increased CS and decreased ZM muscle activation in response to sad facial expressions, by using EMG. Second, we investigated whether avoidant and resistant insecure attachment tendencies, underlying low and high motivation for affiliation, respectively, modulate facial mimicry responses to facial emotional expressions in 3-year-old children. To this end, we tested the hypotheses that children with avoidant attachment tendencies express reduced facial mimicry, whereas children with higher resistant attachment tendencies express enhanced facial mimicry. Furthermore, we hypothesized that the effect of attachment would be stronger for negative affect (i.e. sad emotion), as has been observed in adults [59]. Third, we examined whether selective facial mimicry is influenced by inhibitory control. We hypothesized that the effect of attachment tendencies on facial mimicry would be moderated by inhibitory control.

## Methods

### Participants

Participants were recruited from a database of volunteer families of BLINDED. To broaden the variability of attachment tendencies in our sample, we pre-selected children based on the scores of an online parental attachment questionnaire (see Materials section). Eighty-two children participated in the study, but 40 of them were excluded from the analyses due to technical difficulties with the EMG apparatus (*n* = 14), children not accepting the electrodes on their face or fussiness (*n* = 12), excessive talking and/or movement (*n* = 5), and not reaching the minimum numbers of three trials per condition after artefact rejection (*n* = 9). The criterion set for inclusion was at least 3 trials per condition following previous EMG studies [18, 21]. Such a dropout rate is consistent with previous EMG studies with infants and children [8, 15, 21]. In total, 42 children (25 girls; *M*_*age*_ = 35.26 months, *SD*_*age*_ = 1.17 months, range = 34.2–41.5 months) were included in the final EMG analyses.

Parents whose children completed the EMG recordings successfully were asked to also participate in an attachment home observation. Thirty-seven of the 42 included children were observed at home in a natural and unstructured interaction with one of their parents.

Written informed consent was obtained from all parents prior to participation. Ethical approval for the study was obtained from the Ethics Committee of the Faculty of Social Sciences, BLINDED (ECG2012-1301-006). The study was conducted according to the ethical standards of the Declaration of Helsinki.

### Materials

**Attachment** security was assessed with two instruments: the *Attachment Insecurity Screening Inventory 2–5* (AISI) online parent-report questionnaire [63] and the *Attachment Q-sort* (AQS) home-based observation [64]. While the AISI provides a measure of avoidant and resistant tendencies, the AQS only provides an overall measure of attachment insecurity and was used to test for convergent validity of the AISI questionnaire.

The AISI [63] is a parent-report measure used to assess attachment of children between 2 and 5 years of age. The questionnaire contains 20 items on a 6-point Likert scale, that belong to 3 subscales: avoidant, ambivalent/resistant and disorganized attachment. In line with the hypotheses, only the children’s avoidant and resistant scores were used as independent variables in data analysis. A cut-off score of 46 distinguishes between security (< 46) and insecurity (> 46). Yet, to maximize the variability of scores on avoidant and resistant attachment tendencies, we only considered for participation children with scores < 40 and > 48. Internal consistency analysis yielded Cronbach’s *α* of .80 for the total AISI scale, .74 for the avoidant subscale and .71 for the ambivalent/resistant subscale. According to the AISI cut-off, 57% of children were categorized as secure (< 46) and 43% as insecure (> 46), in the final sample (N = 42). Yet, we only ran the correlation analyses on the total scores of the AISI and AQS, as they are two continuous measures (Table 1). The AQS [64] is a home observation instrument, assessing children between 12 and 48 months of age on attachment security, by sorting 90 items (behaviours) on 9 clusters based on how characteristic or uncharacteristic the behaviour is for the child. Next, the scores of each child are correlated with the scores of a prototypical secure child resulting in a single correlation coefficient that varies between -1 (insecurity) and 1 (security). Each observation session lasted between 90–120 minutes.

**Table 1:** Descriptive statistics and Pearson bivariate correlations among the variables in the study.

	*M(SD)*	*Range*	*Bivariate correlations coefficients*				
			2.	3.	4.	5.	6.
**1. Age (months)**	35.26 (1.17)	34.2–41.5	-.05	.16	-.15	-.32	.15
**2. AISI—Avoidant**	14.10 (3.33)	9–22		.14	.57**	.17	.02
**3. AISI—Resistant**	15.26 (4.73)	8–30			.79**	.03	-.21
**4. AISI (total)**	42.98 (9.18)	26–61				-.01	-.14
**5. AQS**	0.36 (0.16)	0–0.69				-	.06
**6. IC**	3.95 (1)	2–5					-

*Note*. AISI = Attachment insecurity screening instrument; AQS = Attachment Q-sort; IC = inhibitory control. The total sample size for gender, age and AISI scores was 42; the sample size for AQS was 30 and the sample size for IC was 37.

**p < .01.

Although previous findings indicated partial convergent validity between the AISI-avoidant, but not resistant subscale and the AQS [63], in the current study we found no evidence of convergent validity between them (see Table 1). Implications of this finding will be discussed. For the purpose of our investigation of the specific attachment tendencies underlying different motivation for affiliation, namely avoidant and resistant, only the scores for avoidant and resistant attachment tendencies from the AISI questionnaire were used in the analyses.

The **gift delay task** [65] was used to assess children’s inhibitory control. This paradigm requires the child to suppress an immediate response by withholding from a desirable object that was positioned on the table in front of the child. The experimenter explained that he/she had a gift for the child wrapped in colourful paper (width = 21.5 cm, height = 15 cm, depth = 15 cm), but unfortunately forgot the ribbon to close the gift box in another room. Parents were given written instructions about the task and its duration. Through this description sheet, parents were informed that they could choose to go behind a screen with the experimenter or to remain seated at the table with the child and pretend to be busy reading. The experimenter emphasized twice to the child not to touch the box until he/she came back with the ribbon. The lids of the box without the ribbon were slightly loose, such that the child could peek inside, but the gift was not immediately visible. When the experimenter was behind the screen, a timer was set to 3 minutes, while the child was monitored by means of a camera. After 3 minutes, the experimenter returned to the child and closed the box with the ribbon. Eventually the child could open the box and take the gift. Video recordings were coded on a 5-point scale (1 = took the gift from the box; 2 = put the hand in the box; 3 = peeked in the box; 4 = touched the box but did not peek; 5 = did not touch nor peek in the box) [66].

#### EMG paradigm and stimulus material

**Fig 1:**
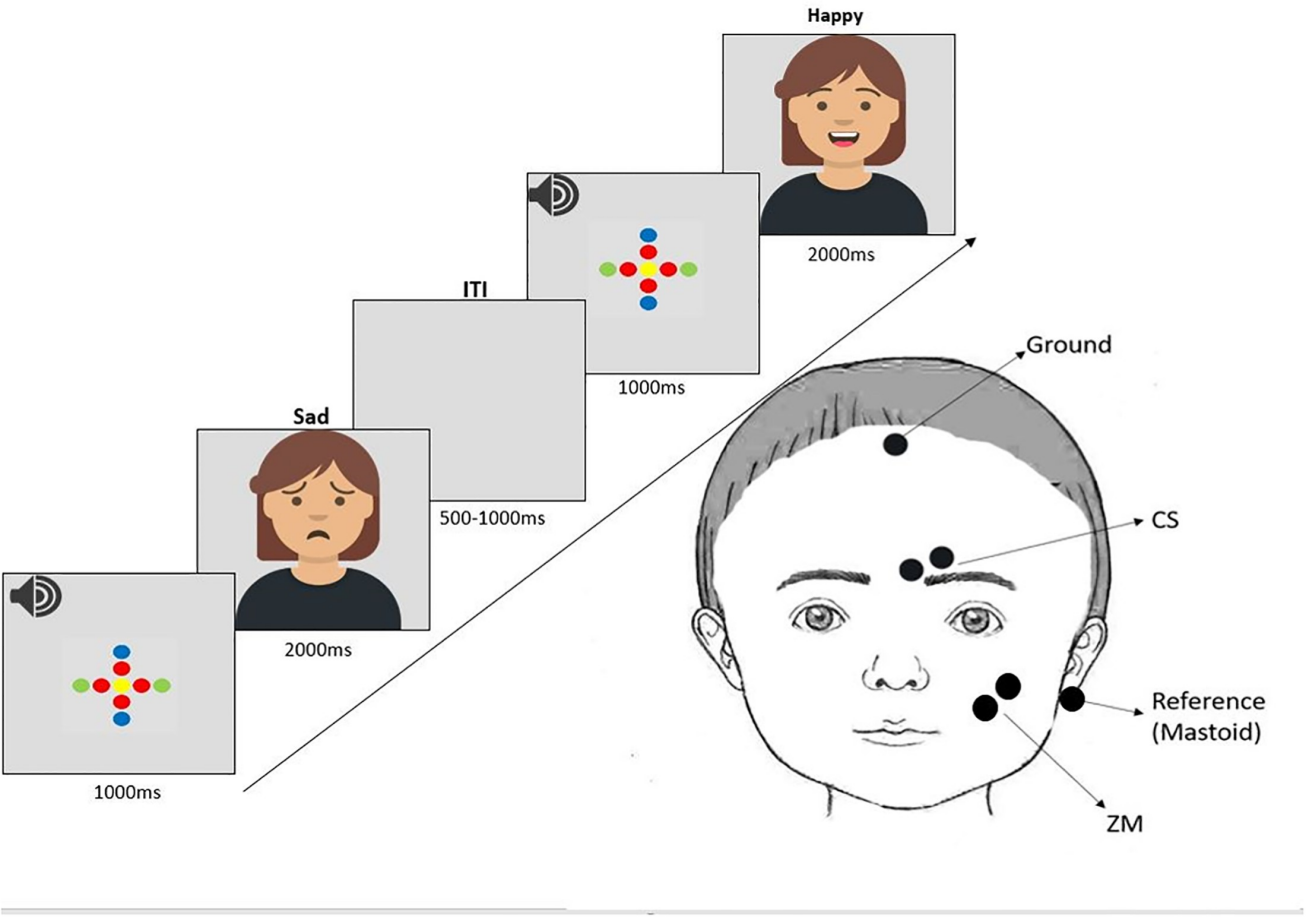
Study design and EMG paradigm. Study design illustrating the trials presentation during facial electromyographic (EMG) recordings over the zygomaticus major muscle (smiling) and the corrugator supercilii muscle (frowning). The example stimuli are not the original images used in the study, due to copyright license of the stimuli from the Radboud Faces Database. The avataars were created and used here for illustrative purposes only (https://getavataaars.com).

For the facial mimicry task, images of facial expressions of happy and sad emotions of white female models were selected from the Radboud Faces Database [67]. A total of six models, expressing both happy and sad expressions were selected from a total of 19 models in a pilot adult study with 10 adult participants, in which we investigated the magnitude of mimicry responses across participants for each condition (happy, sad). The six models that elicited the highest mimicry responses for both happy and sad expressions were selected as stimulus material for the current study, resulting in 12 unique pictures, each repeated ten times. The trials were presented in 5 blocks with each picture repeated twice within one block. Pictures were presented in a pseudo-randomized manner. The presentation lists were prepared with MIX [68] with the following constraints: each model could not be presented more than once consecutively and each emotion could not be presented more than twice in a row. Each trial lasted approximately 4000 ms and unfolded as follows: 1000 ms fixation cross, 2000 ms stimulus presentation and a jittered inter-stimulus interval (ISI) of 500 to 1000 ms. With the onset of the fixation cross a short beep was played as an attention getter. We used an attention getter picture of a cartoon character that could be presented to attract the children’s attention back to the screen if necessary. This attention getter could be initiated through a button press by the experimenter. On average, the cartoon was presented 1.76 times per participant (*SD* = 1.18, *Min* = 0, *Max* = 5) when it was necessary to draw children’s attention back to the screen. The experimenter then waited until the child was calm and had a neutral facial expression, at which point he/she encouraged the child to look at the next pictures. The stimuli were presented for 2000 ms in line with previous adult findings of an effect of attachment on facial mimicry during prolonged presentation of the stimuli [59]. Pictures were displayed on a 17” monitor (1280 x 1024 pixels) and watched from a distance of 60 cm (see Fig 1 for the study design).

#### Procedure

Families were sent an invitation email to fill in the AISI questionnaire via Limesurvey [69]. Upon giving written informed consent, parents filled in the questionnaire and later, selected families were called for an appointment in the lab. All parents who filled in the questionnaire received a thank you letter and stickers for their children.

During the lab visit, children participated in the facial mimicry and the inhibitory control task. The session started with a 10-minute warm-up phase in which children became acquainted with the testing room and the experimenters. Meanwhile, parents were informed about the study and were asked for written informed consent. Additionally, parents received a sheet with a description of the session and instructions for the inhibitory control task. When the child seemed at ease, one experimenter asked the children whether they liked to play with stickers. Then, he/she displayed several cartoon characters (e.g. Mickey Mouse) printed on an A4 sheet, with rectangles on the characters’ faces corresponding to EMG electrodes arrangement. The children were first encouraged to place several stickers on the cartoon character’s face and were then asked whether they would like to have similar stickers on their own face. Then, the experimenter asked the parent and the child to sit in front of a screen (60 cm distance). The child was seated on the parent’s lap and the experimenter explained that they would now receive stickers (i.e. the electrodes) on their face in the same spots as the rectangles in the cartoon sheet. Once the electrodes were applied, the experimenter checked the impedances and the signal quality. When the signal quality was not within the 100 Hz range or impedances were too high, more conductive gel was added to the electrodes and the skin was further cleaned. When the electrodes were well attached, the light was switched off and the experiment started.

Children’s behaviour during the task was recorded with a camera for online monitoring of facial movement and later offline coding of visual attention and movement artefacts. The experiment was interrupted if the child became fussy or did not want to continue. When the EMG session concluded, the electrodes were removed from the children’s face and the face was cleaned with a baby wipe to remove any leftover gel.

The children and the parent moved back to the table where they previously were seated. The lab session was then concluded with the inhibitory control task. At the end of the lab visit, parents were debriefed about the study and were offered either a compensation of 10 euros or a children’s book.

Families of children who performed the EMG task successfully (i.e. accepted the electrodes on their face, attended to most of the stimuli, no technical errors occurred) were called and asked whether they would be willing to participate in the home visit. Upon agreement, an experimenter visited the children and their family at home for the AQS observation. At the end of the observation session, the experimenter debriefed the parents about the study and compensated them for their participation with either 20 euros or two children’s books.

#### EMG recordings and data reduction

EMG responses were measured with Brain Vision Recorder [70]. Pediatric disposable 4-mm Ambu-Neuroline 700 Ag/AgCl surface electrodes were used to record muscle activation from the zygomaticus major (smiling) and corrugator supercilii (frowning) muscles with a bipolar configuration and 10 mm inter-electrode distance between their centres [71,72]. Additionally, the ground electrode was attached on the forehead, below the hairline, and the reference electrode was attached on the mastoid bone, behind the ear. A sampling rate of 2500 Hz was used, and a low cut-off of 10 Hz and a high cut-off of 1000 Hz were applied. To ensure good quality data acquisition, the standard procedures for EMG muscle site preparation and placement were followed [73]. The skin over the muscle group was cleaned and using Nuprep Skin Prep Gel and baby cleanser wipes. Moreover, conductive OneStep clear gel was added to the already pre-gelled electrodes to improve their impedances.

Video recordings of the EMG session were coded offline for visual attention, vocalizations and movement artefacts. Trials in which the children were moving their face or did not attend to the stimuli on the screen during baseline (500 ms pre-stimulus presentation) and during the stimuli presentation (2000 ms) were discarded. On average, children watched 107 trials (*SD* = 20.48) during the experiment and contributed 41.31 (*SD* = 24.92) artefact-free trials, after visual offline coding. Further, EMG data was pre-processed with Brain Vision Analyzer 2.1 [70]. The remaining trials were filtered using a band rejection filter of 50 Hz, 0.2 bandwidth, order 4. Next, an infinite impulse response (IIR) zero phase shift Butterworth filter with a low cut-off frequency of 20 Hz and high cut-off frequency of 500 Hz was applied [74]. After the data pre-processing, artefact rejection based on visual investigation of the EMG signal was conducted. The signals of both muscles were screened between 500 ms before stimulus onset and the 2000 ms of stimulus presentation, hence for segments with a total length of 2500 ms. The segments were inspected for extreme amplitude values outside a 100 mV range. If any peaks during one segment indicated such extreme values, the trial was rejected. The mean number of trials after signal pre-processing that children contributed to the final analysis was 33.36 (*SD* = 26.30, *Min* = 7, *Max* = 119); on average 16 (*SD* = 13.17) for the happy and 17 (*SD* = 13.40) for the sad condition, comparable to previous facial EMG studies with children [8, 23].

#### Statistical analyses

**Fig 2:**
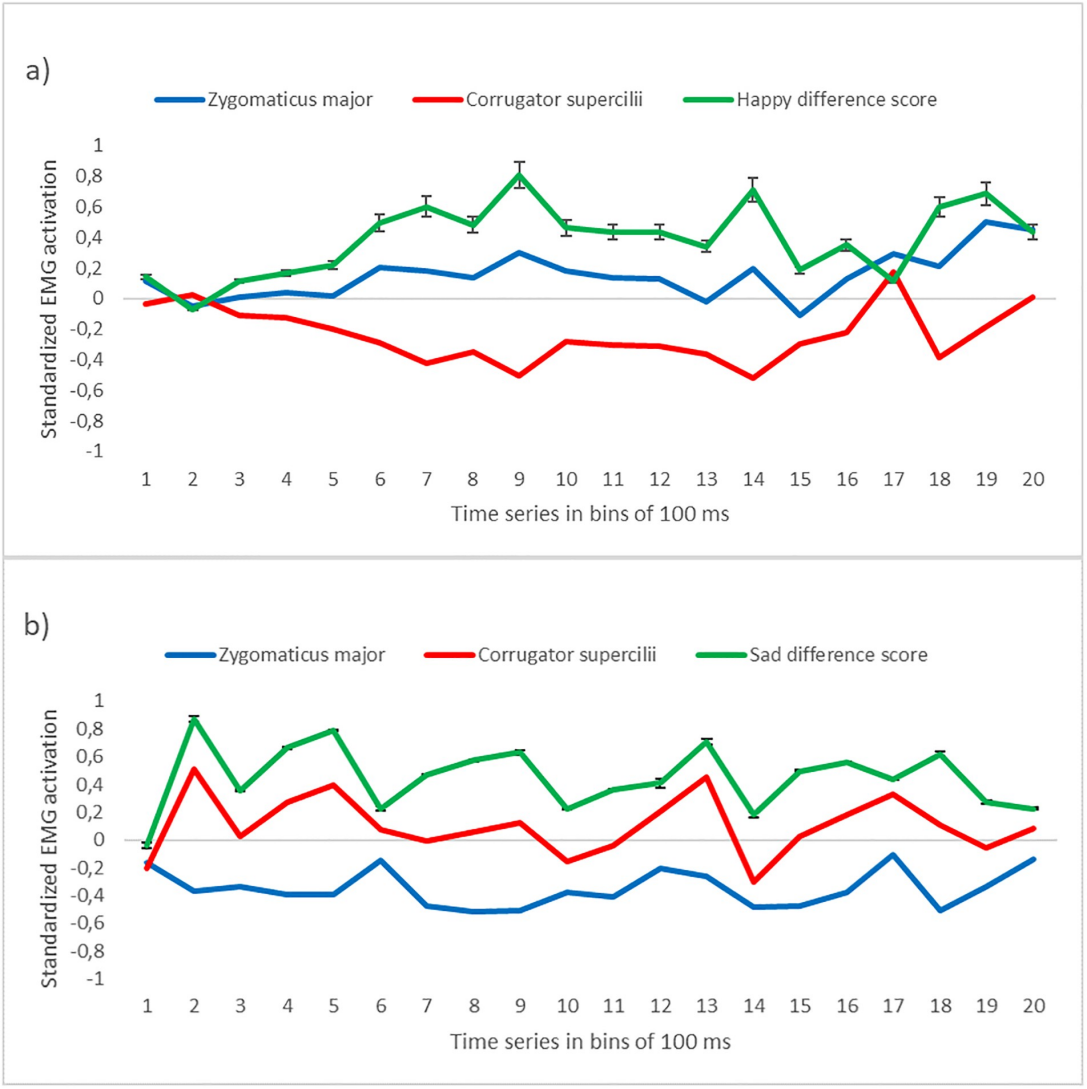
EMG time course activation plots. Time course of standardized EMG activation of the corrugator supercilii (red) and zygomaticus major (blue) muscles, in response to happy (a) and sad (b) stimuli in bins of 100 ms from stimulus onset (0 ms) to stimulus offset (2000 ms). The green line represents the difference score between the two muscles for happy (a) and sad (b) stimuli in which each bin represents the mean (with the standard error) amplitude over 100 ms time.

EMG data was standardized within participants and within muscles to allow comparisons across different muscles [75]. Thus, each 100 ms data bin was standardized by subtracting from each value the mean activation of all the bins and dividing it by the standard deviation of all the bins. Next, we calculated the mean of the baseline bins and performed the baseline correction by subtracting the baseline (1000 ms) mean activation from each 100 ms bin of the time window of in the stimulus presentation (0–2000 ms). For an illustration of the standardized muscle activation of the corrugator supercilii and zygomaticus major muscles for the two different stimuli see Fig 2. After the baseline correction, we calculated the mean activation of the whole 2000 ms time-window for subsequent analyses.

First, prior to testing the research questions, to test whether children displayed facial mimicry in response to happy and sad conditions, we conducted a 2 (Condition: happy, sad) X 2 (Muscle: zygomaticus major, corrugator supercilii) repeated measures ANOVA with EMG activation as dependent variable. Planned posthoc t-test analyses (one-tailed) were performed to test whether—as hypothesized—the zygomaticus major activation was significantly higher than the corrugator supercilii in the happy condition and whether the corrugator supercilii activation was significantly higher than the zygomaticus major in the sad condition.

Second, we tested the research questions, namely whether avoidant or resistant attachment tendencies were related to different levels of mimicry and whether this relationship was moderated by inhibitory control, we ran two hierarchical regression models, one with sad and one with happy facial mimicry as the dependent variables. We computed a mimicry score by calculating the difference scores between zygomaticus and corrugator mean activation for responses to the happy stimuli and between corrugator and zygomaticus for responses to the sad stimuli, similar to previous facial EMG studies [24, 76]. A positive difference score between the zygomaticus and the corrugator represents congruent mimicry responses to happy, whereas a positive difference score between corrugator and zygomaticus indicates a congruent mimicry response to sad expressions.

## Results

Table 1 shows the descriptive statistics of the demographics and the study variables as well as the correlations among them. Unexpectedly, no significant correlations between the AISI subscales and AQS scores emerged (all *p*s > .33).

### Facial mimicry

**Fig 3:**
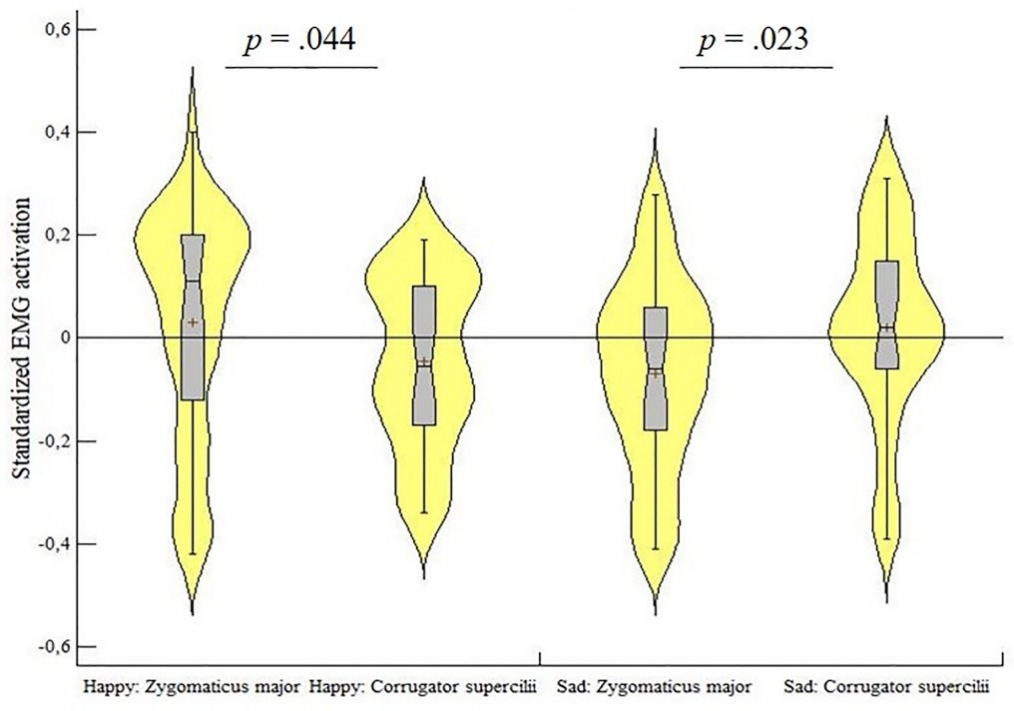
EMG activation violin plots. Violin plots illustrating the standardized EMG activation in the zygomaticus major and corrugator supercilii muscles, in response to happy and sad stimuli. The distribution of the data is represented by the violin shape, with larger width indicating higher value frequency. The mean of each muscle in each condition is represented by a plus sign, whereas the horizontal bars represent the minimum, the median and the maximum values. The whiskers represent the first and the fifth quantile.

One sample t-tests were performed to compare the mean of each baseline-corrected EMG muscle activation for each condition to zero (Table 1 in Supplementary material). The repeated measures ANOVA yielded a significant interaction between condition (happy, sad) and muscle (zygomaticus major, corrugator supercilii) (*F*(1, 41) = 10.35, *p* = .003, *ɳp*^*2*^ = .20), indicating selective activation of the muscles for the corresponding emotional expression (see Fig 3). The activation of the zygomaticus muscle in the happy condition (*M* = .03, *SD* = .22) was stronger compared to the zygomaticus muscle in the sad condition (*M* = -.06, *SD* = 0.17, *t*(41) = 2.66, *p* = .005), indicating that the zygomaticus muscle activation was higher in response to a happy facial expression. Likewise, the corrugator muscle in the sad condition (*M* = 0.02, *SD* = 0.18) was activated more strongly compared to the corrugator muscle in the happy condition (*M* = -.04, *SD* = 0.16, *t*(41) = 2.25, *p* = .015), revealing a specific corrugator activation for sad expressions. Furthermore, a post hoc t-test for the happy condition showed a significantly higher zygomaticus mean activation compared to the corrugator mean activation (*t*(41) = 1.74, *p* = .044), suggesting facial mimicry responses for happy expressions; in contrast, for the sad condition, the corrugator mean activation was significantly higher than the zygomaticus (*t*(41) = 2.04, *p* = .023), suggesting mimicry responses to sad expressions. There were no significant main effects of muscle or condition (all *p*s > .46).

### The relationship between attachment tendencies and facial mimicry and the moderating role of inhibitory control

Two separate hierarchical regression analyses were conducted to investigate whether attachment tendencies (avoidant, resistant), as measured by the AISI, predict facial mimicry responses to sad and happy expressions, respectively. Further, it was investigated whether inhibitory control moderates the relationships between resistant and avoidant attachment tendencies on facial mimicry. Before performing the regression analyses, the predictors were standardized to avoid multicollinearity [77], and three interaction terms were computed: avoidant and resistant attachment tendencies (A X R), avoidant and inhibitory control (A X IC), and resistant and inhibitory control (R X IC).

The first analysis with sad mimicry as dependent variable yielded a non significant omnibus first model of the main predictors (*F*(3, 33) = 2.44, *p* = .082). Nevertheless, resistant, but not avoidant tendencies significantly predicted sad facial mimicry (*β* = 0.35, *t*(3, 33) = 2.14, *p* = .039) (S1 Fig). Moreover, there was a non significant relation between inhibitory control and sad facial mimicry, (*β* = 0.32, *t*(3, 33) = 1.98, *p* = .056). The second model including the interaction terms (A X R, A X IC, R X IC) did not explain significantly more variance (*F*(3, 30) = 0.02, *p* = .841), providing no evidence for the moderating role of inhibitory control in the relationships between avoidant or resistant tendencies and sad facial mimicry.

The second analysis with happy mimicry as outcome variable yielded a non-significant first model of the main effects (*F*(3, 33) = 0.04, *p* = .706) and a non-significant second model of the interaction terms (*F*(3, 30) = 0.15, *p* = .147), suggesting no relationship between attachment tendencies and facial mimicry of happy expressions. The results of the hierarchical regression analyses are summarized in Table 2.

**Table 2:** Hierarchical regression analysis examining the interaction of avoidant (A) or resistant (R) attachment tendencies and inhibitory control (IC) in predicting facial mimicry of sad or happy facial expressions.

	Sad facial mimicry						Happy facial mimicry					
	b	SE	β	*t*	*p*	*(Δ)R*^*2*^	b	SE	β	*t*	*p*	*(Δ)R*^*2*^
**Model 1 (main effects)**					.082	.18					.706	.04
(Constant)	-1.93	1.48		-1.29	.203		0.38	1.66		0.02	.982	
Avoidant (A)	-0.06	0.06	-0.14	-0.92	.361		0.01	0.07	0.04	0.25	.802	
Resistant (R)	0.10	0.04	0.35	2.14	.039*		0.04	0.05	0.13	0.76	.448	
Inhibitory control (IC)	0.45	0.22	0.32	1.98	.056		-0.16	0.25	-0.11	-0.64	.524	
**Model 2 (2-way interaction)**					.841	.02					.147	.19
(Constant)	-1.68	1.62		-1.03	.307		0.41	1.68		0.24	.805	
Avoidant (A)	-0.05	0.06	-0.13	-0.78	.438		0.02	0.71	0.04	0.28	.775	
Resistant (R)	0.07	0.06	0.26	1.24	.223		0.02	0.06	0.09	0.44	.660	
Inhibitory control (IC)	0.45	0.24	0.32	1.89	.068		-0.17	0.24	-0.11	-0.69	.491	
A X R	-0.02	0.27	-0.02	-0.09	.923		-0.50	0.28	-0.36	-1.76	.088	
A X IC	0.09	0.24	0.06	0.38	.702		0.05	0.25	0.03	0.22	.826	
R X IC	-0.22	0.27	-0.15	-0.80	.430		0.51	0.28	0.27	1.82	.079	

Note. *b* = unstandardized regression coefficient; *SE* = standard error; *β* = standardized regression coefficient; *t* = t-value; *p* = significance probability; (Δ)R^2^ = variance explained by the first model and the change in variance explained by the second model. Significance level was set at *p* < .05 (two-tailed):

*p < .05 (N = 37).

Additionally, we conducted an exploratory analysis to investigate whether children who were excluded from the statistical analyses (see Methods section) differed in their attachment scores from children included in the study. To this end, we ran an independent samples t-test investigating the differences in total attachment score (AISI) and the resistant and avoidant subscales. It emerged that children who were excluded (*M* = 16.32, *SD* = 4.99, *N* = 40) scored significantly higher on the avoidant scale (*t*(80) = 2.23, *p* = .021) compared to children who were included in the final analyses (*M* = 14.09, *SD* = 3.33, *N* = 42). No significant differences emerged with regard to the total attachment score and resistant tendencies (all *p*s > .376).

## Discussion

In this study, we investigated whether avoidant and resistant attachment tendencies, underlying low and high motivation for affiliation, respectively, modulate facial mimicry responses in young children, and whether these responses are moderated by inhibitory control. We expected that 3-year-old children would display facial mimicry in response to happy and sad facial expressions, and that attachment tendencies would modulate children’s facial mimicry responses. Specifically, we expected children with avoidant attachment tendencies to display reduced facial mimicry, whereas children with resistant attachment tendencies to show enhanced facial mimicry, especially in response to the sad emotion. Moreover, we proposed that inhibitory control would regulate these responses, namely that children’s enhanced or reduced mimicry would be regulated by their ability to over-express or suppress facial mimicry, depending on their higher (in the case of resistant attachment tendencies) or lower (avoidant attachment tendencies) motivation for affiliation, respectively.

Our EMG findings revealed that children mimicked happy and sad facial expressions, as they displayed higher zygomaticus major activation (smiling) and lower corrugator supercilii activation (frowning) in response to happy facial expressions, and the opposite muscle activation pattern for sad facial expressions. These results are in line with findings by Geangu and colleagues [8] who also showed facial mimicry of happy expressions in 3-year-old children. Importantly, our study provides the first evidence for facial mimicry of sad facial expression in young children. The earliest evidence for sad mimicry so far has been found in children of 6 and 7 years of age [62, 76]. To date, surprisingly few studies have investigated sad facial mimicry even in adults [78–80]. Past research investigating facial mimicry of other negatively valenced facial expressions [8] has tested young children’s mimicry responses of angry expressions, finding evidence for a fear reaction to angry and not mimicry of angry expressions, suggesting that certain emotions (i.e. anger) not only are not affiliative, but produce a reactive response instead. Thus, the finding that children as young as 3-years-old already display facial mimicry of sad expressions is an important contribution to the facial mimicry literature, demonstrating young children’s ability to mimic a number of different emotional facial expressions. Our study and the one by Geangu and colleagues [8] used only female adult models. In order to extend the generalizability of these findings, further investigations should also use adult male and children models.

Furthermore, our data suggested that children with higher resistant attachment tendencies (although within the clinically-secure attachment range) respond with higher sad facial mimicry, suggesting that the higher the motivation for affiliation, the more children manifested mimicry of sad expressions. The interpretation of this result should be exercised cautiously, given that this was the only effect that emerged in the regression model, for which the omnibus test did not reach statistical significance. The finding that resistant attachment scores predicted enhanced sad facial mimicry is in line with our hypothesis that young children employ mimicry selectively, based on their intrinsic motivation for affiliation, and that their intrinsic motivation to affiliate with others and thus facial mimicry is rooted in their early attachment relationships. The specific relationship between resistant attachment tendencies and the mimicry of sad facial expressions could be explained by enhanced attention and hypervigilance in response to cues in the environment that could possibly be threatening (e.g. negatively valences emotional expressions) [81]. Indeed, Fraley and colleagues [81] found a heightened perceptual sensitivity to emotional cues in anxiously, but not avoidant attached individuals, and slightly stronger sensitivity for sad than happy emotional expressions. In addition, previous studies have shown an increased sensitivity to faces [82] and enhanced neural activation in areas linked to facial mimicry (i.e. orbitofrontal cortex) in anxiously attached individuals [83].

Interestingly, and contrary to our hypothesis, avoidant attachment tendencies did not yield a suppression of facial mimicry, although a pattern in the expected direction was observed. Evidence from an adult study [59] showed that individuals characterized by avoidant attachment suppress facial mimicry responses, particularly in response to negative facial expressions. Interestingly, we found a significant difference in avoidant scores between children who were excluded from the analyses (e.g. not accepting the electrodes on their face, fussiness, excessive talking, not enough trials) and children who provided sufficient data to be included in the statistical analyses. This finding suggests that children characterized by avoidant attachment tendencies were more likely to refuse the electrodes on their faces or to become fussy during the experiment. This lack of children characterized by high avoidant attachment in our sample might also be one of the reasons why we did not find a statistically significant modulation of facial mimicry by avoidant tendencies. These findings provide important insights into the feasibility of investigating insecure attachment in relation to highly sensitive measures, such as facial EMG. Accordingly, future research should cautiously consider drop-out rates in relation to insecure attachment and take this into account for the study design. Moreover, the results also did not provide evidence for the regulatory role of inhibitory control on selective facial mimicry. This finding is in contrast to that of van Schaik and Hunnius [33], showing that inhibitory control predicts selective behavioural mimicry in children. As those children were older than the children in our study, it could be the case that the role of inhibitory control becomes more prominent during later childhood. Furthermore, it is noteworthy that in contrast to the former study, that measured a more explicit form of mimicry (i.e. behavioural mimicry), in our study we measured an implicit form of mimicry. However, the lack of effects of avoidant attachment tendencies and inhibitory control highlight an alternative argument.

It could be argued that interindividual differences in the motivation for affiliation, as stemming from early attachment relationships are not subject to a controlled suppression of mimicry responses, but rather reflects a learned response that individuals employ. Indeed, infants spend a substantial amount of their awake time in face-to-face interactions with their caregivers, and thereby, bearing in mind that attachment tendencies stem from these early interactions [84], facial mimicry could be an acquired communication skill that individuals manifest throughout the life-span. A study comparing two samples of 5- to 10-year-old maltreated and control children showed that maltreated children displayed altered facial mimicry responses and in particular suppressed corrugator supercilii activation to negative facial expressions [85]. These responses might thus be an early functional adaptation to the environment. The suppressed mimicry response to negative facial emotional expressions by maltreated children is in line with the findings by Sonnby-Borgström and Jönsson [59], suggesting mimicry suppression to negative facial emotional expressions by insecurely avoidant individuals, at a subliminal level, possibly due to emotional regulation difficulties. Based on early experiences and interactions, young children might learn that displaying or mimicking certain expressions and not others is more advantageous and functional in their environment. Importantly, though, our sample was drawn from the general population which was not characterized by clinical insecure attachment categories, but rather by avoidant and resistant attachment tendencies. Thus, it may be that we could not optimally capture strong interindividual differences in their motivation for affiliation, or the lack of it. Future studies are needed to investigate these differences, for example in a context that explicitly activates the attachment system, triggering avoidant or attachment tendencies in children’s behavioural responses, or within clinical samples.

Furthermore, children’s attachment tendencies were evaluated in relation to their primary caregivers, while facial mimicry was measured towards unfamiliar others. Early attachment patterns are known to generalize to other relationships during later development [85], and as such we assessed attachment as a proxy for one’s affiliative motives with others. Nevertheless, it could be the case that the attachment effects on facial mimicry would be stronger in response to caregiver’s facial expressions, in line with the proposition that facial mimicry may emerge within the context of face-to-face interaction between parents and their infants [13]. Future research should further explore the interrelation between attachment tendencies and facial mimicry towards primary caregivers and unfamiliar others. Finally, we found no evidence for the modulation of happy mimicry by different attachment tendencies. This could be due to the fact that happy expressions are readily mimicked from early infancy [23]. Happy expressions indicate pleasant interactions and thus individuals might not need to modulate their responses to these. On the contrary, mimicking sad emotions may be emotionally costly for children characterized by insecure attachment, as this emotion signal distress. Altogether, our findings together with those by Sonnby-Borgström and Jönsson [59] in which the modulation of attachment emerged specifically for sad and angry facial mimicry, respectively, demonstrate that insecurely attached individuals are particularly sensitive to negative affect.

One limitation that requires attention in the interpretation of these results is the assessment of attachment. Interestingly, contrary to previous evidence on the partial convergence validity between AISI and AQS [63], we found no significant relations between the two instruments. Both instruments separately yielded attachment scores that are comparable to previously reported scores in the general population [63]. Furthermore, while the focus of the AQS is on the child-parent interaction and the quality of this relationship, that of the AISI is more on the children’s behaviours as reported by their parents. Although this can provide an explanation of the lack of convergence between the AISI and the AQS, it does not explain why we do not replicate the findings by Wissink and colleagues [63, 86]. More data is needed to examine the relation between the AISI and the AQS further.

In summary, our study provides evidence for facial mimicry of positive and negative affiliative emotional expressions in 3-year-olds. Moreover, children’s higher affiliation motivation (resistant attachment tendencies), but not reduced affiliation motivation (avoidant attachment tendencies), were found to modulate facial mimicry for sad expressions. Altogether, these findings open a new window of investigation of how early interactions shape later social behaviours.

## Supporting information

S1 Fig Scatterplot and regression line illustrating the relation between resistant attachment scores and sad facial mimicry. On the y-axis the standardized EMG facial activation for sad facial expressions is displayed, while on the x-axis the standardized resistant attachment scores are plotted.
(TIFF)

S1 Table Facial EMG activation in response to happy and sad facial expressions. *Note*: *M*—mean; *SD*—standard deviation, *t*—value of one sample t-test (one-tailed) comparing mean value for each muscle to zero. EMG muscle activation was previously baseline corrected and z-standardized within muscles for each condition and within participants.
(DOCX)
