# Correction: The modulation of facial mimicry by attachment tendencies and their underlying affiliation motives in 3-year-olds: An EMG study

**DOI:** 10.1371/journal.pone.0225493

**Published:** 2019-11-14

**Authors:** Stefania V. Vacaru, Johanna E. van Schaik, Sabine Hunnius

The name of the institution where the participants were recruited from and the name of the institution where informed consent was obtained from have been incorrectly withheld from the published article.

In the Participants subsection of the Methods, there is an error in the first sentence of the first paragraph. The correct sentence is: “Participants were recruited from a database of volunteer families of the Baby and Child Research Center Radboud University, the Netherlands.”

In the Participants subsection of the Methods, there is an error in the second sentence of the third paragraph. The correct sentence is: “Ethical approval for the study was obtained from the Ethics Committee of the Faculty of Social Sciences, Radboud University, the Netherlands (ECG2012-1301-006).”
