# Supplementary figures and images for: The modulation of facial mimicry by attachment tendencies and their underlying affiliation motives in 3-year-olds: An EMG study
Source: PLoS One. 2019 Jul 1;14(7):e0218676. doi: 10.1371/journal.pone.0218676 (PMC6602198; doi:10.1371/journal.pone.0218676)

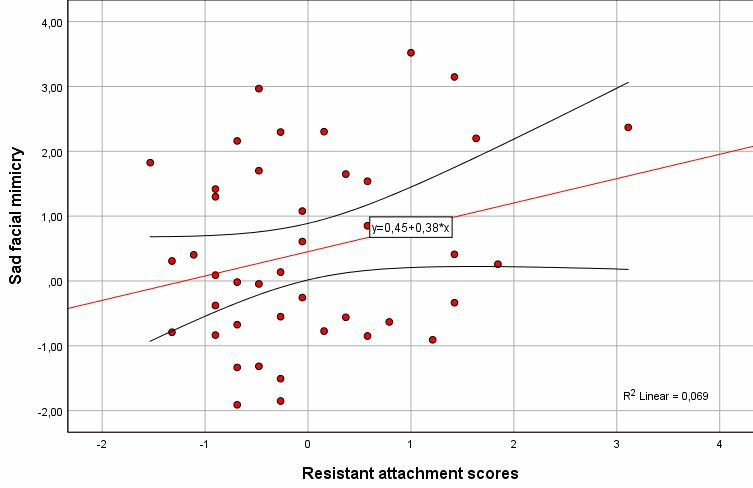

Supplement: S1 Fig — On the y-axis the standardized EMG facial activation for sad facial expressions is displayed, while on the x-axis the standardized resistant attachment scores are plotted. (TIFF) [file pone.0218676.s001.tiff]
