# Supplementary material for: The modulation of facial mimicry by attachment tendencies and their underlying affiliation motives in 3-year-olds: An EMG study
Source: PLoS One. 2019 Jul 1;14(7):e0218676. doi: 10.1371/journal.pone.0218676 (PMC6602198; doi:10.1371/journal.pone.0218676)
Supplement: S1 Table — Note: M—mean; SD—standard deviation, t—value of one sample t-test (one-tailed) comparing mean value for each muscle to zero. EMG muscle activation was previously baseline corrected and z-standardized within muscles for each condition and within participants. (DOCX) [file pone.0218676.s002.docx]

**S1 Table. Facial EMG activation in response to happy and sad facial expressions**

| Presented emotion | EMG muscle site | *M* | *SD* | *t* | *p* |
| --- | --- | --- | --- | --- | --- |
| **Happy** | ZM | 0.15 | 1.11 | 0.89 | .188 |
|  | CS | -0.23 | 0.81 | -1.85 | .035 |
| **Sad** | ZM | -0.35 | 0.89 | -2.50 | .008 |
|  | CS | 0.10 | 0.91 | 0.74 | .231 |

*Note:* M - mean; SD - standard deviation, t - value of one sample t-test (one-tailed) comparing mean value for each muscle to zero. EMG muscle activation was previously baseline corrected and z-standardized within muscles for each condition and within participants.
